# Autotherapy in Abdominal Infections

**Published:** 1917-07

**Authors:** Charles H. Duncan

**Affiliations:** New York City


BUFFALO MEDICAL JOURNAL

Yearly Volume 72 JULY, 1917 Number 12

### ORIGINAL ARTICLES

**The right is reserved to decline papers not dealing with practical medical and surgical subjects, and such as might offend or fail to interest readers. Contributors are solely responsible for opinions, methods of expression and revision of proof.**

---

### Autotherapy in Abdominal Infections.

By CHARLES H. DUNCAN, M. D., New York City.

In autotherapy the physician treats the patient with the unmodified toxic substances developed within the latter's body, by the action of the infecting agent on his body tissues, against which the tissues react in a curative manner.

The autotherapeutic remedy is usually obtained by filtering the pathogenic exudate through a Duncan Autotherapeutic Apparatus. The filtrate contains the bacteria-free unmodified toxin-complex, or the toxic result of germ activity, from **all of the microorganisms in the locus of infection,** in the same proportion and virulence in which they appear in the patients body. The immunizing filtrate is injected hypodermically.

The Doctrine of Aggressins, developed by Bail, need only to be referred to, and not outlined here, to cause those who are familiar with the subject of immunity to understand the action of these injected toxins. There is no danger in their use, or no more danger than attends the use of the vaccines and sera now in daily use among us. Autotherapy is indicated wherever a vaccine is indicated. The patient suffering with an infection, has the remedy designed by nature to cure him within his body, and it often can be obtained; for it is the unmodified toxic substances within the patients body, that the tissues must resist in establishing a spontaneous cure.

During the winter of 1909 and 1910 the writer began treating patients suffering with infections with the unmodified toxic substances developed within the locus of infection by the action of the infecting agent on his body tissues. In the regular order of development of this new system of therapy, he treated several cases of acute appendicitis, cholecyctitis, etc., successfully by filtering the sputum and injecting the filtrate hypodermically, and published his results.

These cures were severely attacked from many quarters, for as yet, there was no precedent for such deep seated infections being cured so quickly, and that by such simple means. Since then, Dr. Edward C. Rosenow of the Mayo's clinic, has shown by actual tests on animals many reasons why it is possible for these cures to be made. Rosenow's tests show that bacteria have an elective affinity or trophism for the tissues upon which they grew. Thus, cultures of streptococci taken from a human appendix have a strong tendency to develop appendicitis in animals when injected intravenously. When the strains are cultured for a week or longer but few of the animals develop appendicitis. Cultures from **tonsils** of individuals having appendicitis will tend to develop appendicitis cholecyctitis, etc., respectively in animals when injected intravenously, and microscopically he identified the same causative microorganism in both the appendix and tonsils, furthermore he demonstrated the same microorganism present in the tonsils after the operation for appendicitis was performed. Thus, it is shown that operation does not remove the cause of the disease, but merely some of the effects. It is the condition behind and deeper than the operation that autotherapy often removes. This statement is apparently borne out by further tests by Rosenow. He shows that even the **bacteria-free filtrate** from cultures of the streptococcus taken from an inflamed appendix markedly affect by elective affinity the appendix of animals into which they are injected. It is altogether probable that filtrates from the exudates without cultivation would be more positive in their action. Rosenow states—"Streptococcus loose in characteristic elective affinity by cultivation on artificial media, after animal passage, etc.," Then it logically follows that they should not be so grown, if the best therapeutic results are to be obtained; and that the ideal curative agent in one that is cultivated upon no media, except the patients own body tissues, the elective affinity of these toxins remains unaltered by filtration. The autotherapeutic remedy is the only strictly autogenous agent we have at our command in fighting disease. In view of the above tests we must accept the autotherapeutic cures of acute appendicitis, peritonitis, etc., as reported, for they have now been experimentally demonstrated. These tests lays at rest forever the criticism of autotherapy that these cures could not be made for the reason there has been no precedent for them. The pain of acute appendicitis, cholecyctitis, peritonitis, etc., will usually subside within a few hours after the first injection, as if by the action of morphine, and what is more it will not return if a few more injections are given according to the needs of the patient.

In an article under the title of "Autoimmunization in Respiratory Infections" that appeared in the **Medical Record** September 5th, 1914, after reporting two cases of acute appendicitis and two cases of cholecyctitis cured quickly by injecting the filtrate of sputum subcutaneously, the writer stated: "If the physician treats catarrhal conditions of the respiratory tract autotherapeutically, he will often be surprised tô discover he has cured many conditions supposed to be remote from this locality." This holds true for a wide range of infections. By following the technic of my former articles many physicians are injecting the filtrate of mucus from the respiratory tract and curing infections of, the mastoid cells, the antrum of Hymore, the frontal sinuses, bronchitides acute and chronic, tonsilitides, (it is very important that this latter disease be treated autotherapeutically, for rheumatism and heart complications so often follow the usual vegetable and mineral drug medication for this disease), laryngitides, pharyngitides, etc., quickly. Diseases of the eye that have their origin, in infections of the nares, should be treated autotherapeutically; this last statement includes glacoma before which the physician is so often helpless.

In autotherapy we are not immunizing the patient to the socalled autoginous vaccine, for this is of altered or lowered therapeutic value compared to the unaltered toxins or the autotherapeutic remedy; nor are we immunizing our patient to microorganisms taken from some other patient or source, grown on innumerable cultures. In autotherapy we immunize the patient to his own unaltered toxins, that is toxins from all of the microorganisms, both causative and complicating, present in the locus of infection. This is what nature does when a spontaneous cure occurs, autoimmunizes the patient. With this understanding we can readily see that if a vaccine cures, such cure is due, not to the laboratory cultures, preserving chemicals, heat, etc., but in spite of them. The question then is asked: Should the infecting microorganisms ever be cultured for therapeutic purposes? Microorganisms should never be grown on culture media as we now grow them, if it is done with the view of adding to their therapeutic effect.

Case 1. Patient, female, age 22 years, was taken to Belleview Hospital where she was operated for acute appendicitis. She was dicharged ten days later when she returned to work. Within five days she came down with severe abdominal pains. The writer was called and found her suffering with acute peritonitis. She was given an injection of the filtrate of sputum at 5 o'clock p. m. She slept the greater part of the night, the pain having left in six hours. She had three other similar injections; there has been no return now for eleven months.

It is not intended in this brief article to do more than stimulate interest in this new method of therapy; but still the technic is so very elementary simple and the results so positive that it will repay any one to be prepared to treat these deep infections, for by this means he will be able to abort many operations that formerly demanded operation. The literature should be examined carefully, but if the following technic is closely followed it is possible to cure many cases. Whether an operation is performed, autotherapy should be employed as an additional factor of safety, for surgical operation simply removes some of the effects of the infection, while autotherapy tends and often does remove the cause.

Mix one drachm of sputum with an ounce of distilled water. Allow it to stand for twenty-four hours, (in an emergency twelve hours will often suffice), with occasional agitation. After which time filter through a Duncan Autotherapeutic Apparatus and inject from one to one and a half cubic centimeters subcutaneously. Disperse the injected fluid through the tissues by vigorous rubbing through a sterile bandage. The pains will usually leave within twelve hours. Do not repeat the injection for five days unless the pain returns. Then inject again. The cutaneous reaction is manifested by a pink discoloration of the skin adjacent to the point of the needle. Within wide range, it may be stated the greater the cutaneous reaction the quicker will be the response and cure. The constitutional reaction is manifest at times by a slight chill. This is due to the size of the dose being larger than the local tissues can take care of. If a severe chill folows do not repeat the injection till ten days has elapsed since the last, unless the pain returns. The return of pain is the indication for another dose. The above technic is for acute conditions. In chronic conditions, very much smaller doses should be employed, and the interval between doses may have to be increased. The range of dosage is very wide, so that no fear need be entertained if the technic outlined above is closely followed.

It may be stated in closing that the above technic is applicable to all infections that have their origin in infections in the respiratory tract. Both cutaneous and constitutional reactions will usually subside within twenty-four hours.

### REFERENCES.

1. Edward C. Rosenow: Elective Localization of Streptococci. Journal of the American Medical Association, November 13, 1915.
2. Edward C. Rosenow: An Epidemic of Appendicitis, and Parotitis Probably Due to Streptococci Contained in Dairy Products. The Journal of Infectious Diseases, April, 1916.
3. Edward C. Rosenow and Sverre Oftedal: The Etiology and Experimental Production of Herpes Zoster. From the Memorial Institute of Infectious Diseases and The Mayo Foundation. Reprint.
4. Charles H. Duncan: Autoimmunization in Respiratory Infections . Medical Record, September 5, 1914.
5. Charles H. Duncan: Autotherapy. New York Medical Journal, December 14 and 21, 1912.
6. Charles H. Duncan: Autotherapy versus Operation. The American Practitioner, July, 1913.
7. Charles H. Duncan: Elective Localization of the Streptococcus from an Autotherapeutic point of view. New York Medical Journal, February 10, 1917.

---

**Foreign Bodies in Hydrocele.** Stuart Graves reports in the Miss. Valley Med. Jour., June, a routine autopsy on a negro aged 75. 3 faceted bodies, about 1 c.m. in diameter were found in an old right hydrocele, one having been palpated before section. Organized fibrin was found on section so that the term foreign body is not strictly applicable, nor is it stated that calcification was observed, so that the term concrement is scarcely applicable. Reference is made to a peritoneal formation, of the size of a fist, of similar nature, from McGill University. Such bodies seem to be analogous to those sometimes found in joints, especially the knee and known by the term Gelenkmaus.

---

**Reduction of Typhoid in Massachusetts.** The Public Health Bulletin reports a rate of 4.5 per 100,000 population for 1916, the lowest rate for any state yet recorded. It is only a few years since a rate of 15-20 was considered low for a state or city and, even in Europe, a rate of 10 has been considered normal. The progress is ascribed largely to the increased use of antityphoid injection, sanitary measures having already achieved their maximum practically attainable effect.

